# Skin conditions in diabetes mellitus: a clinical practice based panel

**DOI:** 10.1186/1758-5996-7-S1-A31

**Published:** 2015-11-11

**Authors:** Luis Clemente Rolim, Marco Andrey Cipiani Frade, Maria Regina Calsonari, Samanta Nunes, Helena Schmid, Geisa Macedo, Miguel Nasser Hissa, Karla Freire Rezende, Luiz Fernando Andrade Feijó, Tania Barreto

**Affiliations:** 1 Sanofi Aventis, São Paulo, Brazil

## Background

Dermatologic conditions, such as dry skin and callus, are very frequent in patients with diabetes mellitus (DM) and, in fact, increase the risk of important outcomes, as skin lesions, ulcerations, and diabetic foot. Patient, nurse, and physician education are essential steps to prevent and better understand DM patient flow regarding skin conditions and its possible outcomes.

## Objective

To describe lack of information regarding dermatologic conditions in DM from a physician's perspective, and also to suggest new strategies to address this subject.

## Materials and methods

A 16-questions questionnaire regarding dermatologic conditions in DM patients was developed and sent to academic specialists (six endocrinologists and two dermatologists), from different geographic locations in Brazil. Subsequently, a panel discussion with all the respondents was performed to validate the responses. During the panel, additional relevant data were collected. The questionnaire and the panel discussion were representative of physician's clinical practice.

## Results

The specialists had mean clinical experience of 25 yrs., with the mean patient volume of 62 patients/month per physician, achieving 496 patients/month. According to questionnaire responses and the panel discussion, skin conditions are present in at least 55% of DM patients. In fact, all participants classified dermatologic conditions in DM as crucial or important, emphasizing that early diagnosis of skin pathologies can avoid the evolution to worse prognosis, such as skin lesions, subsequent infections, diabetic foot, and amputations. In addition, 62.5% of the specialists considered treating dry skin an important step to prevent diabetic foot complications and 50% of the specialists considered hydration an important additional care to DM patients. Also, physician's and patient's education regarding dermatology conditions were emphasized by all the specialists.

## Conclusions

According to the compiled data from the questionnaire and panel, the lack of information is a crucial issue regarding dermatologic complications in DM patients, due to unrecognized importance of early-stage diagnosis. Simple and common conditions like dry skin can progress to severe dermatological outcomes. In fact, the specialists considered nurses', physicians', and patients' education and early identification and treatment of skin problems in diabetics the main strategies to improve this clinical condition.

